# Prioritising in a crisis: what next for malaria research?

**DOI:** 10.1186/s12936-026-05922-z

**Published:** 2026-04-25

**Authors:** E. G. Chestnutt, M. O. Afolabi, U. D’Alessandro, M. Delves, R. Gosling, G. Jagoe, E. Lasry, C. Maiteki, R. W. Moon, E. Oriero, C. Pitt, F. Tadesse, J. K. Tibenderana, V. B. Rao

**Affiliations:** 1 Malaria Consortium, London, UK; 2 Malaria Centre, London School of Hygiene and Tropical Medicine, London, UK; 3 Faculty of Infectious and Tropical Diseases, London School of Hygiene and Tropical Medicine (LSHTM), Keppel Street, London, UK; 4 Medical Research Council Unit The Gambia at the London School of Hygiene and Tropical Medicine, Fajara, The Gambia; 5 Department of Disease Control, Faculty of Infectious and Tropical Diseases, London School of Hygiene & Tropical Medicine, Keppel Street, London, UK; 6 Medicines for Malaria Venture, Geneva, Switzerland; 7 The Global Fund to Fight AIDS, Tuberculosis and Malaria, Geneva, Switzerland; 8 National Malaria Control Division, Ministry of Health, Kampala, Uganda; 9 Global Health Department, Faculty of Public Health and Policy, London School of Hygiene & Tropical Medicine, London, UK; 10 Armauer Hansen Research Institute, Addis Ababa, Ethiopia; 11 Médecins Sans Frontières, London, UK

**Keywords:** Malaria

## Abstract

Global malaria control is entering a period of profound uncertainty, with earlier gains now stalling or reversing amid a rapidly deepening shortfall in programme and research funding. At the same time, parasite and vector resistance, changing transmission patterns driven by climate and urbanisation, and growing conflict and displacement are reshaping malaria epidemiology and undermining the effectiveness of core tools. In this context, there is an urgent need to reconsider how malaria research is prioritised, funded and conducted. Drawing on a diverse group of stakeholders, this Perspective argues that malaria research must adopt a dual mandate: protecting lives now while preparing programmes for the next generation of biological and systemic threats. We highlight three interlinked priorities. First, pragmatic, equity‑focused implementation and health‑systems research should be prioritised to optimise the effectiveness, efficiency and coverage of existing interventions, including through integrated delivery models and closer collaboration with affected communities and implementers. Second, sustained investment in innovation—such as next‑generation chemoprevention strategies, vaccines and monoclonal antibodies, novel vector control approaches, and digital or AI‑enabled surveillance—is essential, accompanied by operational research that addresses feasibility, cost and pathways to scale in real‑world conditions. Third, we highlight a critical research stream on sustainable and diversified financing, and on how data, economic evidence and political economy analysis can more effectively inform national and global decision‑making. By re‑orienting towards an impact‑driven, context‑sensitive and value‑for‑money research agenda, the malaria community can help stabilise fragile programmes, reduce the risk of resurgence and preserve momentum towards long-term malaria elimination in an era of constraint.

## Background

Global malaria control stands at a critical juncture. After two decades of progress, momentum has stalled, and in some regions reversed. In 2024, there were an estimated 282 million malaria cases and 610,000 deaths, with Africa bearing 95% of the mortality burden [1]. The sudden withdrawal of major donor funding in 2024 and 2025 has exposed the vulnerability of health systems, forcing countries to make stark choices between maintaining essential services and investing in innovation. Concurrently, intensifying biological threats, climate shocks, and humanitarian crises are reshaping malaria epidemiology and threatening recent gains.

At this inflection point, malaria research must fulfil a dual mandate: safeguard lives now and future-proof programmes against emerging challenges. (1) We ignore research at our peril but priority should be given to research that strengthens the effectiveness, efficiency, and equity of current interventions, while fostering evidence-driven adaptation to maximise impact amid constrained resources. (2) Continued investment in innovation, including next-generation prevention tools, improved diagnostics, and digital surveillance, is vital given malaria continues to our understanding and our evolving toolbox. (3) We cannot afford to ignore implementers and communities living with malaria: pragmatic, operational research embedded in programmes and co-designed with communities ensures scalable, context-relevant solutions designed for real-life use. (4) Investigating sustainable financing mechanisms is crucial to stabilise programmes and enable rapid deployment of lifesaving interventions.

Drawing on the insights of a World Malaria Day 2025 expert panel and recent evidence, this Perspective advocates for an impact-focused research agenda that addresses urgent questions, demonstrates value-for-money, and maintains momentum towards long-term elimination goals.

## Reality check: reductions in funding across all sectors likely to remain

The recent cuts to global health funding are having a dramatic impact on the lives of some of the poorest communities in the most challenging contexts. As early as 2017, WHO warned about the widening gap between the resources required to achieve the Global Technical Strategy for malaria (GTS) and the funds actually allocated [2]. Since then, the gap has continued to grow, with major donors reducing spending on overseas development assistance as their focus pivots to domestic challenges following global economic shocks including the COVID-19 pandemic [3]. In 2023, globally malaria programmes were already experiencing a 57% shortfall in funding to meet GTS targets with 50% of funding coming from major donors including the USA, UK, France, and Germany [4]. However, 2025, has marked a year where what was once a plateau in funding is now a substantial decline. The dismantling of the President’s Malaria Initiative removed both financial support and valuable technical expertise [5]. With other major funders following suit, the paradigm for how we pursue malaria reduction and elimination has shifted profoundly. This is likely to be sustained, and applies across all sectors beyond malaria.

These funding cuts are already disrupting malaria programmes through workforce shortages, supply chain failures and weakened outbreak preparedness and surveillance [6, 7]. We know from experience that previous abandonment of malaria elimination programmes in the 1960’s quickly led to decades of avoidable malaria resurgence. Stepping back today leaves us at risk of a generational setback.

Similarly, malaria research and development funding has remained below the long-term average, even prior to recent funding cuts. The three largest funders of research in 2024 were the US National Institutes of Health (NIH) (27%, US$ 197.3 million), aggregated industry (25%, US$ 180.5 million) and the Gates Foundation (24%, US$ 174.5 million) with the UK FCDO increasing its contribution to US$ 38.7 million (5%). This money was mainly spent on drug development and basic science research [1]. If the proposed NIH cuts are fully realised, then malaria research funding soon will be primarily reliant on philanthropy and industry. This changing landscape raises important questions regarding who will be setting the research agenda and for whose benefit.

## Reality check: emerging threats challenging our toolbox

This rapid cessation of programmatic and research funding has come at a time of converging and emerging threats. These include biological threats which reduce the ability of current tools to prevent, diagnose and treat malaria, where research is urgently needed.

For example, the malaria parasite has evolved in many regions through the loss of histidine rich protein 2/3 (HRP2/3) genes. These genes produce the protein detected by the most distributed malaria rapid diagnostic tests (RDTs), causing false-negative results leading to untreated malaria. Similarly genetic mutations responsible for drug resistance can reduce the effectiveness of first line treatments to rapidly clear infections, allowing onward transmission of partially resistant strains of malaria. If not addressed programmatically, these mutations can lead to treatment failure, requiring different drug regimens, such as multiple first line therapies, to respond.

The need for new tools and formulations is not limited to the parasite. Vector control is the foundation of malaria control. Yet, insecticide resistance has been documented in almost all endemic countries, and mosquitoes are adapting their behaviour—biting outside and earlier—which circumvents many existing tools designed for vectors that bite indoors and at night. Invasive mosquitoes and climate change are also reshaping transmission dynamics in new geographies. As a result, urban and highland areas, which were once viewed as lower risk settings, are now experiencing increases in malaria transmission necessitating new control strategies and products.

Rising numbers of conflicts and humanitarian crises, which are also intricately entwined with climate vulnerability, result in displacement which increases malaria risk and reduces access to malaria and other health services. Evidence of these threats are already visible in several countries. Ethiopia, a country that was once highlighted as a success in malaria control having reduced cases to below one million in 2019, has faced a confluence of threats resulting in a resurgence to over 10 million cases in 2024 [3].

## Research is critical but needs to remain relevant

These emerging threats, coupled with reductions in funding, have created a perfect storm of escalating risk and diminishing response capacity. As researchers and practitioners of global health, we must recognise the imperative need to re-prioritise and refocus research and implementation efforts, to address these threats but also to remain relevant, with malaria-affected communities central. Recognising the complexity of balancing immediate priorities with long-term vision, we suggest practical ways the malaria community must adapt considering the evolving funding landscape. These include evaluating cost-effective integrated delivery strategies to guide the immediate restructuring of malaria programming, and researchers and funders making a sustained commitment to innovation-focused research. Continued investment is essential to address both emerging and future challenges, ensuring the goal of malaria eradication is not forgotten amidst current political upheaval and burgeoning threats.

### A coordinated approach for effective use of resources

This new era provides an opportunity to improve efficiency by breaking down silos which were once necessary to secure malaria funding in a donor-driven financing landscape. An integrated approach, focusing on overall health outcomes, can optimise the use of expertise and resources, address gaps, and minimise duplication. But such an approach needs research to ensure integrated programmes are effectively and equitably realised, while safeguarding the gains made in malaria up to today.

#### Reimagining delivery approaches with equity

An immediate opportunity for research is identification of more efficient ways to implement existing interventions with current systems and fewer resources. However, such analyses need appropriate research on equity considerations to avoid a ‘maximising impact’ strategy resulting in prioritising high intervention coverage in fewer areas or uncomfortable trade-offs such as, accepting lower coverage of prevention strategies, in favour of less resource-intensive delivery mechanisms. However, since malaria disproportionately affects the poorest and most disadvantaged populations, research must support partners to create and refine strategies that maximise impact while prioritising equity.

#### Collaborating across and beyond the health sector

Additionally research must demonstrate how collaboration can improve effective delivery and reduce duplication. Integrated campaigns that include seasonal malaria chemoprevention (SMC), bed nets and, better coordination between malaria, HIV and maternal and child health programmes can help increase coverage of antenatal care, malaria prevention and prevention of mother-to-child transmission. Integrating supply chain approaches across programmes, and with the private sector, can create increasingly necessary efficiencies. As these approaches are developed, they must be evaluated to assess what works best for replication and scale—and what does not. Including feedback loops, such as community-led monitoring, can help ensure access is maintained.

Looking beyond the health sector can support improved collaboration with ministries of finance, education and environment can contribute to more creative approaches and finding fruitful synergies. This requires malaria researchers to also embrace collaboration with other disciplines to analyse models of coordination.

Furthermore, ministries of finance, the private sector, and non-traditional donors require data that demonstrate the importance and value of investing in malaria relative to the competing demands on their resources. Unlike case counts alone, metrics such as disability-adjusted life years (DALYs) and years of life lost (YLL) quantify malaria’s health burden in ways that can be compared with other diseases and health areas. However, to use such evidence decision-makers also need clear, accessible explanations tailored to non-technical audiences, which are timely and align with their priorities. Collaborating with economists to embed cost, budget impact, and cost-effectiveness analyses within implementation research can equip decision-makers with the evidence required to assess trade-offs and allocate resources effectively. Political economy analysis can help to identify the decision-makers to provide evidence to. Researchers should also work closely with decision-makers to align research outputs with fiscal and policy cycles, ensuring relevant evidence is available ahead of key decision-making moments.

#### Tracking and sharing data

A third action, available to all in the research community is to track, document and share information more widely. Pooling data can support decision-making for resource allocation, increase impact and support advocacy. Mechanisms for knowledge sharing and learning, such as open data platforms like MalariaGEN [8], can facilitate global data dissemination that can be used to inform incremental cost-effectiveness ratios and identify which interventions are most effective in different contexts. Data are already being used to identify how to achieve the highest impact with limited resources in Uganda where mass distribution of long-lasting insecticidal nets has been reduced in urban areas to enable dual-AI nets, which are more expensive, to be deployed in areas where they can have the greatest impact [9]. Data and information sharing can also help to identify challenges quickly and mobilise a coordinated response.

### Futureproofing and preparing for our next challenges

Research is critical not only to protect the interventions we have today and extending our armamentarium as much as possible, but also to develop the models of care and delivery, prevention, diagnostics, treatment and even financing mechanisms of tomorrow. At the same time, research methodologies need to be embedded in implementation in simple ways that can help sustainable monitoring of interventions, and provide timely answers to decision-makers, otherwise, they will continue to be at risk of being deprioritized. The malaria research community is uniquely placed to link innovation to implementation, enabling testing not only whether new tools work, but how they can be delivered equitably, affordably, and at scale.

#### Expanding existing tools

One pragmatic pathway to futureproof programming is to identify existing malaria interventions that can be expanded to cover new age groups and geographies to enhance disease control and drive progress towards elimination. A notable example is the evolution of the World Health Organization guidance on SMC. Originally, SMC was recommended primarily for children aged 3–59 months in areas with highly seasonal malaria transmission. However, ongoing research and operational evidence have demonstrated the benefits of extending SMC to new regions and older children where malaria seasonality and transmission patterns warrant broader protection. These findings have informed changes in WHO guidelines, enabling programme implementers to adapt SMC delivery to local epidemiological realities [10]. Such adaptive approaches, grounded in rigorous research and aligned with community needs, are essential for maximising the impact of malaria interventions and ensuring no risk groups are left behind.

#### Novel tools, technologies, and delivery

A second way is to invest in innovations that can improve feasibility and reduce cost of delivery. New tools already in the pipeline offer hope for more effective prevention, diagnosis, and treatment. Among the most promising tools under development are next-generation interventions such as long-acting injectables, single-dose cure, second-generation vaccines, and monoclonal antibodies like L9LS, which have demonstrated high protective efficacy across malaria seasons and where possible offer minimum burden delivery schedules or address specific, often neglected needs including pregnant women and neonates [11]. Novel vector control methods, including genetically modified mosquitoes, are also showing promising results [12]. In addition, research into digital technologies, including predictive modelling, anticipatory action, and AI-driven surveillance, is opening new avenues to optimise resource allocation and maximise the impact of interventions. These innovations, while potentially transformative, often present challenges related to cost and logistics, underlining the need for inventive programming, operational research, and co-design with communities to ensure their successful and sustainable deployment [13]. By integrating these new tools with patient-centred approaches and resilient health systems, there is significant potential to accelerate progress towards malaria elimination.

Innovative approaches to the delivery of malaria interventions can also support cost reduction while maximising uptake among target populations. Recent research has investigated whether consolidating vaccine doses into a single vial, rather than multiple vials, can streamline logistics and lower expenses, simplifying distribution and administration in resource-limited settings [14, 15]. These efforts underscore the importance of operational research and co-design with end users to ensure new delivery methods not only enhance efficiency but also facilitate greater reach and impact for malaria control programmes.

#### Investing in new financing models

A third opportunity is to focus research on strengthening the evidence base on how malaria programmes can be financed more sustainably. One notable example is the International Finance Facility for Immunization, which mobilises funds by issuing bonds on capital markets, thereby frontloading resources for the rapid rollout of vaccines and other lifesaving tools [16]. Additionally, the use of insurance schemes, direct facility funding and social contracting approaches are emerging as promising mechanisms to promote sustainable financing, allowing for more predictable and flexible funding streams that can adapt to evolving needs. These financial innovations, when combined with robust operational research and cross-sectoral collaboration, enable malaria programmes to overcome traditional funding limitations and address challenges related to cost and logistics, ultimately supporting the effective deployment of next-generation tools and interventions.

## Relevance and reality

The dismantling of existing global health financing has understandably been met with widespread anxiety. Although the challenges are great and the situation is complex, change can also create the opportunity to do something differently. Recently, there has been some reinstatement of funding from the US Government albeit at diminished levels, which provides a fragment of hope for malaria programmes. Research must shift to the new reality, and the evolving situation raises important questions about who will shape the direction of future research and who will benefit from it. To mitigate the risks of underinvestment in malaria, as a research community need to understand how we stay relevant. We must work collaboratively, pursue innovation and ensure resources are invested efficiently for meaningful outcomes. By supporting research aligned with ground-needs, fostering strategic partnerships and investing in new tools we can secure the foundation for future malaria eradication.

## Data Availability

Not applicable.
